# Patient Safety in Anesthesiology: Progress, Challenges, and Prospects

**DOI:** 10.7759/cureus.69540

**Published:** 2024-09-16

**Authors:** Wafaa Harfaoui, Mustapha Alilou, Ahmed Rhassane El Adib, Saad Zidouh, Aziz Zentar, Brahim Lekehal, Lahcen Belyamani, Majdouline Obtel

**Affiliations:** 1 Epidemiology and Public Health, Laboratory of Community Health, Preventive Medicine and Hygiene, Faculty of Medicine and Pharmacy, Mohammed V University, Rabat, MAR; 2 Epidemiology and Public Health, Laboratory of Biostatistics, Clinical Research and Epidemiology, Faculty of Medicine and Pharmacy, Mohammed V University, Rabat, MAR; 3 Intensive Care Unit, Hospital Ibn Sina, Rabat, MAR; 4 Faculty of Medicine and Pharmacy, Cadi Ayyad University, Marrakesh, MAR; 5 Mohamed VI Faculty of Medicine, Mohammed VI University of Health Sciences, Casablanca, MAR; 6 Faculty of Medicine and Pharmacy, Mohammed V University, Rabat, MAR; 7 Emergency Unit, Mohammed V Military Hospital, Rabat, MAR; 8 Direction, Military Nursing School of Rabat, Rabat, MAR; 9 General Surgery, Mohammed V Military Hospital, Rabat, MAR; 10 Vascular Surgery, Ibn Sina University Hospital Center, Rabat, MAR; 11 Mohammed VI Foundation of Health Sciences, Mohammed VI University, Rabat, MAR; 12 Royal Medical Clinic, Mohammed V Military Hospital, Rabat, MAR

**Keywords:** anesthesiology, artificial intelligence, challenges and inequalities of access, future prospects, low and middle-income countries, patient safety, progress in anesthesiology, technological advances

## Abstract

Anesthesiology is considered a complex medical specialty. Its history has been marked by radical advances and profound transformations, owing to technical and pharmacological developments and innovations in the field, enabling us over the years to improve patient outcomes and perform longer, more complex surgical procedures on more fragile patients. However, anesthesiology has never been safe and free of challenges. Despite the advances made, it still faces risks associated with the practice of anesthesia, for both patients and healthcare professionals, and with some of the specific challenges encountered in low and middle-income countries. In this context, certain actions and initiatives must be carried out collaboratively. In addition, recent technologies and innovations such as simulation, genomics, artificial intelligence, and robotics hold promise for further improving patient safety in anesthesiology and overcoming existing challenges, making it possible to offer safer, more effective, and personalized anesthesia. However, this requires rigorous monitoring of ethical aspects and the reliability of the studies to reap the full benefits of the new technology. This literature review presents the evolution of anesthesiology over time, its current challenges, and its promising future. It underlines the importance of the new technologies and the need to pursue efforts and strengthen research in anesthesiology to overcome the persistent challenges and benefit from the advantages of the latest technology to guarantee safe, high-quality anesthesia with universal access.

## Introduction and background

Anesthesiology has undergone remarkable evolution and revolutionary progress, with its origins dating back thousands of years to the first experiments using traditional anesthetic substances. However, the early days of anesthesia were marked by numerous risks, and the practice was viewed as dangerous, with frequent incidents and complications. For instance, the mortality rates were 1 in 2,000 to 2,500 with chloroform and 1 in 25,000 with ether [1].

However, the fields of surgery and anesthesiology have evolved collaboratively [2]. Without advances in anesthesiology, surgical advances would not have been possible [3,4]. Furthermore, the emergence of less invasive surgical techniques has not only made anesthesiologists’ work easier [5] but it has also led to a reduction in the need for blood transfusions [6]. Moreover, the specialty of anesthesiology has continued to evolve thanks to the pioneers of anesthesiology who have made a significant contribution to the development and improvement of anesthetic techniques through the deepening of anatomical knowledge, the introduction of new anesthetic agents, the use of more precise administration methods, the use of more secure and reliable surveillance and monitoring technologies, and more sophisticated equipment. The creation of professional associations and societies to ensure continuous professional development and the establishment of national and international guidelines and recommendations have made anesthetic practice a medical specialty today [7]. It has revolutionized contemporary medicine, allowing quality surgery, complex interventions, a significant improvement in the safety and quality of anesthetic care, and a notable reduction in risks [8]. As a result, some studies have shown that mortality has fallen dramatically, from 6.4 deaths per 10,000 operations in the 1940s to 0.4 per 100,000 at the end of the 1980s [9]. Today, the risk is even lower, with fewer than one death for every 200,000 to 300,000 anesthetic procedures performed [10].

Although modern anesthesiology has made considerable progress, it faces complex and multidimensional challenges. On the one hand, there are risks inherent in anesthesiology, such as the potentially serious side effects of drugs and complications associated with anesthetic techniques and equipment. On the other hand, factors and characteristics specific to each patient, such as their state of health and medical history, as well as the variability of individual response, further increase the complexity of anesthetic management. In addition, anesthesia professionals face significant constraints, such as high workload, burnout, and the risk of legal action, which can impact the quality of care and patient safety. In addition, in low- and middle-income countries, there are specific challenges related to unequal access to care, limited resources, a shortage of qualified staff, and a lack of continuing education, which hinder progress toward safe and affordable anesthesia care [11,12]. To tackle the existing problems, some actions and initiatives need to be carried out collaboratively, at the technical, human, and organizational levels, while complying with recommended standards.

Today, promising innovations and new emerging technologies can revolutionize anesthetic practice by opening up exciting new ways to improve safety and efficiency in anesthesiology by offering innovative solutions to recurring challenges by integrating simulation, a teaching and assessment method that uses artificial, interactive scenarios to reproduce clinical situations. These techniques can replace or enhance real patient experiences with guided experiments designed to evoke significant elements of the real setting [13], which can enhance the professional learning and development of anesthesiologists. Artificial intelligence (AI) refers to techniques that enable computers to imitate human intelligence, relying on algorithms to solve problems and make decisions. It includes machine learning (ML) and deep learning (DL) [14], which can optimize monitoring the depth of anesthesiology, ensure pain management, automate the administration of anesthetics, predict adverse events, and plan and organize the operating theater. Furthermore, robotic anesthesia systems have also been developed to help anesthesiologists with various tasks, reduce medical errors, and provide remote access to expertise. Genomics, which studies the genetic variability of the response to drugs and aims to understand interindividual pharmacological variations [15], has also opened up new prospects for personalized, targeted anesthesia, helping anticipate the risk of complications. However, the new technologies face several challenges and ethical considerations that need to be addressed to reap the full benefits, such as the lack of quality data and algorithmic biases that could compromise the reliability of diagnosis and treatment. In addition, there are crucial ethical issues surrounding the confidentiality of health data, liability in the event of medical error, and equity of access to these technologies, particularly given their high cost. In addition, the absence of a clear and appropriate legal framework represents a further obstacle to safe and responsible use [16,17].

This literature review aims to present a general understanding of safety in anesthesiology and evaluate the results of existing research on the various aspects related to this medical practice. We conducted an extensive and in-depth search of various databases, including PubMed, ScienceDirect, Web of Science, Scopus, and Google Scholar. The keywords chosen were related to patient safety in anesthesiology and related outcomes. Specifically, we used search terms such as “advances in anesthesiology” “challenges,” and “emerging innovations.” The literature search did not set a date limit and comprised publications up to 2024. We included data from literature reviews, systematic reviews, meta-analyses, original research articles, books, and book chapters. Additionally, we incorporated findings from publications and websites from the World Health Organization and various national and international professional associations of anesthetists. We examined the abstracts and then evaluated the full text of the documents, making it possible to systematize the results into three main parts. The first examines the historical development of anesthesiology, focusing on the technological and pharmacological advances and safe practices that have been achieved. The second identifies the main problems persisting to the present day related to anesthesiology, which require optimal and rapid interventions to be overcome. The third explores the role of new advances that are more precise and effective, as well as promising tools offering opportunities to improve patient safety and ensure high-quality, safe, and affordable anesthesia care while addressing existing challenges. Rigorous implementation is essential to carefully assess their limitations, challenges, and ethical implications to derive their full benefits which are likely to revolutionize the practice of anesthesia worldwide in the near future.

## Review

Historical development

The history of anesthesiology has been marked by significant evolution since its origins, with constant progress in the field of anesthesia leading to optimal patient safety over the years.

The Evolution of Anesthetic Agents

Modern anesthesiology is just over a century and a half old, dating back to antiquity, with the empirical use of natural substances such as opium, alcohol, and certain plants, or mechanical techniques such as nerve compression [18]. During the 17th and 18th centuries, anesthesiology progressed thanks to greater knowledge of the anatomy of the heart and lungs, as well as a better understanding and discovery of gases. Horace Wells succeeded in using nitrous oxide to extract teeth in 1844, William Thomas Green Morton proved in 1846 that ether could be used as an anesthetic [19], and James Simpson discovered chloroform in 1847 [20]. However, the tragic death of Hannah Green in 1848 highlighted the potential dangers of this new substance. Subsequently, John Snow conducted extensive studies of the new anesthetics, discovering that chloroform was stronger than ether [21]. Subsequently, the introduction of modern volatile anesthetics, more powerful and better controlled, led to significant progress. Halothane, a non-flammable halogenated agent, was adopted in the 1950s, and enflurane was marketed in 1973; however, they are virtually no longer used because of their undesirable effects [22]. Indeed, the most common volatile anesthetics since the 1980s-1990s are isoflurane, desflurane, and sevoflurane, offering better pharmacology and greater safety than their predecessors [23]. Inhalation anesthesia was the norm until the appearance of thiopental in 1934, a barbiturate used for the induction and maintenance of general anesthesia [24], followed by etomidate, an agent with stable hemodynamic effects. Subsequently, ketamine, which produced a particular anesthetic state, and propofol, developed in the 1970s, came to the fore thanks to their stable hemodynamic effects and rapid recovery [25]. On the other hand, dexmedetomidine was introduced as a novel intravenous anesthetic adjuvant, providing sedative, analgesic, anxiolytic, sympatholytic, and opioid-sparing properties, while maintaining a stable hemodynamic effect [26]. In addition, the introduction of curare in 1942 marked a significant advancement in anesthesia, making endotracheal intubation easier, optimizing surgical conditions, and assisting mechanical ventilation in patients with reduced pulmonary compliance. Their use has considerably simplified airway management and made it safer while reducing complications [27,28]. Since then, various neuromuscular agents, such as suxamethonium, pancuronium, vecuronium, atracurium, rocuronium, mivacurium, and cisatracurium have been developed, each offering varied pharmacological properties to adapt to the specific needs of patients [29]. However, some agents, such as rapacuronium, have been withdrawn from the market because of significant side effects, including an increased risk of bronchospasm [30]. Furthermore, the development of opioids has seen significant advancements, with the discovery of fentanyl in 1950, followed by new analogs such as alfentanil and sufentanil, aimed at slightly modifying pharmacological properties, and then remifentanil, with its even briefer effect [31,32], despite an increased risk of dependence associated with their high liposolubility and rapid onset of action [33].

The Development of Anesthesia Administration Techniques

Intravenous administration of anesthesia was made possible by the arrival of the Pravaz syringe in 1852 [34]. Subsequently, for better control of anesthesia, researchers such as Thomas Skinner developed the first masks and inhalers for anesthesia [35]. Further, thanks to the work of Bouchut, O'Dwyer, Macewen, and others, tracheal intubation also evolved, followed by the cuffed endotracheal tube, which revolutionized patient safety during surgery [1]. However, before this, laryngeal examinations in 1855 were conducted using mirrors. The invention of rigid laryngoscopes and reflective laryngoscopes marked a step forward, but it was Macintosh’s direct laryngoscope developed in 1943 that truly innovated laryngeal visualization [36-38]. However, the Guedel cannula became an essential tool in anesthetic practice. Moreover, these advancements led to an evolution in anesthesia [39]. On the other hand, the origins of local anesthesia date back to the 19th century, when William Halsted used cocaine to perform nerve blocks, and in the early 20th century, new techniques were developed, including the axillary brachial plexus block by Kulenkampff in 1911 and the sciatic nerve block by Labat in 1922 [40,41]. The 1940s-1960s saw the emergence of new, more effective local anesthetics, in parallel with advances in nerve localization techniques, improving the quality and duration of locoregional anesthesia [42]. Since then, locoregional anesthesia has occupied a central place in anesthetic management, making it possible to reduce the consumption of general anesthetics, reduce postoperative pain, and accelerate patient recovery. Moreover, techniques have diversified and are often the alternative of choice to general anesthesia [43].

The Revolution in Sophisticated Equipment

Numerous advances have been made thanks to the development of anesthetic equipment, contributing to the evolution of modern airway management and anesthesia techniques [44]. In addition, the beginnings of mechanical ventilation date back to the 16th century, with the first attempts at bellows resuscitation [45]. Subsequently, new ventilation methods were adopted, such as forced respiration in the case of deep anesthesia, and positive pressure ventilation for artificial respiration in the case of apnea. Indeed, early anesthesia systems lacked today’s safety features, forcing anesthetists to be extremely vigilant to avoid the risks associated with potentially dangerous defects. Later, major advances came with the invention of the spirophore by Voyer in 1876, followed by Heinrich Dräger’s pulmotor, the forerunner of pneumatic ventilators [46]. Indeed, the addition of a soda lime cartridge without valves enabled better control of carbon dioxide levels. However, problems with lines and connectors and shortcomings in equipment design sometimes led to the accidental administration of hypoxic mixtures, with tragic consequences [47]. Hence, in the 1950s, gas machines were equipped with ventilators. Currently, non-invasive ventilation with continuous positive airway pressure, bilevel positive airway pressure, and high-flow nasal cannula oxygenation has been used. These techniques have demonstrated effectiveness in reducing the need for postoperative intubation and the amount of sedatives given to prevent endotracheal tube dislodgement [48]. However, significant progress has also been made in integrating surveillance and alarm systems [49].

On the other hand, anesthesia monitoring standards evolved with the integration of the electrocardiogram, highlighting its role in the early detection of arrhythmias and cardiac abnormalities [50]. Pulse oximetry has become the reference method for detecting respiratory disorders [51], further complemented by capnography to identify early abnormalities [52], along with the use of non-invasive blood pressure measurement, temperature monitoring, and neuromuscular monitoring. In addition, current recommendations cover a wide range of physiological parameters, from cardiovascular and respiratory monitoring to processed electroencephalogram monitoring and metabolic, to ensure optimal patient care in the pre, per, and postoperative phases [53].

Improving Patient Safety in Anesthesiology

Ever since the first anesthetic procedures were performed, anesthesiologists have been aware of the risks involved and have worked to ensure patient safety and prevent adverse events. Indeed, the Second World War had a major impact on the field of anesthesiology. On the one hand, the lack of equipment stimulated innovation, and, on the other, the shortage of nursing staff necessitated the rapid training of many anesthesiologists and subsequently led to a standardization of training, reinforcing the importance of this specialty [54].

Patient safety in anesthesia has been identified as the set of measures taken to minimize anesthesia-related risks and guarantee patient safety. In addition, numerous efforts have been made to significantly improve safety through pharmacological and technical improvements, followed by the development and widespread adoption of practice guidelines [10]. In particular, the introduction of halogenated anesthetics marked a turning point in anesthetic practice, offering significant advantages in terms of safety, efficacy, and control [55]. In addition, the use of pentothal demonstrated a significant drop in the mortality rate, from 1 in 450 to 1 in 5,500 between 1943 and 1944 [56]. At the same time, more precise administration techniques, such as locoregional anesthesia, helped to limit complications [57]. Second, monitoring equipment has improved considerably, enabling more detailed monitoring of the patient’s vital parameters. In addition, emphasis has been placed on the continuing education of anesthetists and the adoption of protocols, national and international safety recommendations, and the development of standardized checklists [58,59]. Furthermore, since the early 20th century, professional associations and societies have played an essential role in the field of anesthesia, bringing professionals together, promoting best practices, advancing research, and ensuring patient safety, with the 1905 founding of the Society of Long Island Anesthetists, followed by the creation of the Society of Anesthesiologists of New York. The American Society of Anesthesiologists became the leading organization in 1936 [60], supporting the creation of the American Board of Anesthesiology in 1937. The research-oriented International Anesthesia Research Society helped found the World Federation of Societies of Anesthesiologists in 1955 [61,62]. Today, multiple associations in the field of anesthesia have emerged and remain key players, organizing congresses and editing publications that contribute to the advancement of this medical specialty [63].

Current status and challenges

Today, modern anesthesia faces complex challenges linked to the intrinsic risks of the anesthetic act, patient characteristics, constraints on healthcare professionals, as well as problems of access to care, and lack of resources and staff training, particularly in low- and middle-income countries.

Risks Associated With the Anesthetic Procedure

Anesthesia has always been known for its side effects and specific complications. Indeed, incidents correspond to avoidable events linked to the administration of anesthesia, which may have or have had harmful results for the patient. On the other hand, despite advances in anesthesiology, its minor side effects and common complications remain without significant change, such as nausea and vomiting, which can affect up to 30% of patients [64], dry mouth, sore throat, muscle pain, itching, chills, drowsiness, dental damage, and peripheral nerve damage [65]. In addition, long-term sequelae or life-threatening complications [66] may occur, such as respiratory complications (hypoxia, bronchospasm, and inhalation) due to airway obstruction, difficult intubation, or allergic reaction to anesthetics, in addition to cardiovascular complications (hypotension, arrhythmias, and cardiac arrest), especially in patients with heart disease, and neurological complications (neuropathy, stroke and postoperative confusion), can result from inadequate perfusion of the brain or a reaction to anesthetic drugs. In addition, serious drug-related complications such as allergic reactions and dosage errors can occur [67]. Indeed, studies have shown that allergic reactions, although rare, can be potentially fatal, occurring in 0.1-0.01% of cases under general anesthesia [68]. In addition, intubation-related complications such as intubation failure, esophageal intubation, pulmonary inhalation, and hypoxia have been frequently reported. According to one study, peri-intubation cardiac arrest occurred in 4% of emergency patients, with a hospital mortality rate of 84%, even after the return of spontaneous circulation [69]. In contrast, cardiac arrest associated with general, regional, and local anesthesia has decreased over time [70].

The main complications of locoregional anesthesia include toxicity, which can occur with excessively high doses, particularly in frail patients, along with neurological complications such as nerve damage. In addition, the risk of infection and catheter displacement requires special attention, and serious respiratory complications, such as apnea, have also been observed in some cases [71]. In addition, malfunctions of anesthesia equipment and devices can lead to serious intraoperative complications. At present, although the malfunctions previously reported in the literature have become less likely with the new generations, unexpected problems can still occur [72]. Therefore, it is essential to pay particular attention to the selection and administration of anesthetics to minimize the associated risks. Indeed, rigorous anesthetic management, based on proven protocols and guidelines, is crucial to ensure patient safety and prevent serious complications [73]. Furthermore, neglect of the checklist in the operating theater can have serious consequences for patient safety, and the absence of systematic checks can lead to errors that should never occur. The use of checklists has proven to be effective in reducing morbidity and mortality, prolonged stays, and re-hospitalization. Therefore, the checklist is highly recommended in anesthesia, as it is an essential tool for improving patient safety [74]. In addition, several epidemiological studies have shown that inadequate preoperative preparation can be a major factor contributing to perioperative mortality [75], while better organization of perioperative care can provide high-quality patient care. On the other hand, anesthetic gases pose a threat to the environment and public health, contributing to greenhouse gas emissions that contribute to global warming. Yet, measures such as low-flow anesthesia, regional techniques, and total intravenous anesthesia (TIVA) can reduce this impact. Furthermore, volatile agents can also present a potential health risk to operating theater staff if improperly managed and disposed of. The search for sustainable alternatives and raising awareness among healthcare professionals are therefore crucial to minimizing the ecological footprint of anesthesia [76,77].

Patient-Related Risks

Many of the risks associated with anesthesia are closely linked to the patient’s condition. Indeed, certain health factors and medical histories such as obesity, advanced age, smoking, cannabis use, and sleep apnea can increase the risks associated with anesthesia [78]. In addition, medications taken regularly by the patient are likely to interact with anesthetic agents and alter the patient’s response, necessitating an adjustment in management [79]. In addition, preoperative stress and anxiety can hurt the patient’s physiological response to anesthesia and increase the risk of complications. In this context, medical teams must take the time to inform and reassure patients about the anesthetic procedure. Indeed, patients’ apprehension and lack of understanding of anesthesia can exacerbate their stress reactions, with potential repercussions on hemodynamic stability, sensitivity to anesthetic agents, or the occurrence of postoperative complications [80]. Furthermore, some studies have shown that anesthesia plays a significant role in the course of cancer patients, influencing the progression of tumor cells and the risk of recurrence by modulating the immune system and inflammatory response. Indeed, volatile anesthetics can promote the growth of cancer cells, while propofol and regional anesthesia appear to offer protective effects. In addition, TIVA may improve recurrence-free survival. However, further studies are needed to better understand the impact of the choice of anesthesia on patients’ long-term prognosis [81,82]. Certainly, a patient’s medical history has a considerable influence on the choice of anesthetic technique, yet a thorough preoperative consultation helps to assess the patient’s general state of health and to adapt anesthetic management, significantly reducing the risk of complications [83].

Risks Associated With Healthcare Professionals

Anesthesiologists face significant challenges, and several factors are likely to harm the quality of care and safety of anesthetic management of patients [84,85]. Indeed, psychological and emotional factors among anesthetists, such as anxiety or burnout, can influence their practice and ability to properly manage complications [86]. In addition, fatigue and work overload can lead to a decrease in psychomotor performance, communication, and teamwork skills, as well as an increase in medical errors and the risk of adverse effects for patients [87]. However, limited human performance, such as staff incompetence in the face of the situation, decision-making errors, inappropriate technical actions, data confusion, negligence, or failure to follow good practices are all risks that can lead to adverse events [88]. In addition, fear of legal action [89] seems to be a major factor influencing anesthetists’ behavior. Medical malpractice complaints lead some experienced doctors to perform unnecessary tests or even abandon the practice, while for trainee doctors, these complaints hinder their professional development and lead them to adopt defensive medical practices to protect themselves [90]. Certainly, actions and initiatives are crucial to manage professional stress, prevent burnout, and mitigate the risk of lawsuits to guarantee safe, quality anesthetic care. In addition, good work management and organization can effectively prevent staff burnout.

Challenges Specific to Low- and Middle-Income Countries

Low- and middle-income countries suffer from a major disparity in anesthetic safety. Indeed, several studies have revealed significant differences in anesthesia-related mortality rates from one country to another [91,92]. Although developed countries have succeeded in significantly reducing anesthesia-related mortality rates to as low as 2 per 10,000 to 1 per 200,000 in healthy patients, this is unfortunately not the case worldwide. Indeed, in many low- and middle-income countries, particularly in Africa, anesthesia-related mortality rates remain much higher, as high as 1 in 300 [62]. Moreover, maternal mortality after cesarean section is 50 times higher than in developed countries [93].

Yet, the WHO warns that 5 billion people in developing countries, over a third of the world’s population, do not benefit from safe, affordable surgical care and anesthesia and that the distribution of anesthesiologists is extremely uneven, with fewer than 5 per 100,000 inhabitants in 77 countries and fewer than 1 per 100,000 in 43 countries [94,95]. Table 1 illustrates the various challenges faced by developing countries concerning anesthesia, with these shortcomings contributing to higher rates of complications and mortality in these regions [96,97]. First, there is a crucial lack of financial resources, as well as inadequate infrastructure and equipment. In addition, these countries face a critical shortage of qualified personnel, accompanied by a lack of motivation among professionals. Second, their healthcare systems are fragile, and the provision of anesthesia care is inadequate, leading to wide disparities and limited access to care. Finally, the lack of continuing education is a major obstacle to the development of anesthesia in these regions.

**Table 1:** The main challenges facing anesthesiology in low- and middle-income countries.

Challenges	Description
Insufficient financial resources and infrastructure	Lack of resources, specialized personnel, specialized equipment, and funding [98]. Limited health budgets, reducing investment in equipment, drugs, and training [99]. Inadequate and poorly maintained healthcare facilities (equipment, monitors, drugs, etc.) [97]. High cost of anesthetic agents and specialized equipment [100]
Shortage of qualified personnel and lack of motivation	A severe shortage of qualified anesthesiologists [95]. Low- and middle-income countries, accounting for 48% of the world’s population, have only 15% of anesthetists, and the majority of anesthesia is performed by non-physicians [101]. Low remuneration and recognition with difficult working conditions make retention of qualified staff difficult [102]
Fragile health systems and inadequate healthcare provision	Lack of appropriate hospital infrastructure (operating theaters, intensive care units, equipment maintenance) [99]. Perioperative mortality rates are high in many countries, with around 15% of all deaths attributed to poor-quality healthcare. In addition, 60% of preventable maternal deaths and 53% of deaths in children under five are due to inadequate healthcare [86]. Inadequate coordination and management of anesthesia services, with gaps in quality of care, notably in referral circuits, early diagnosis and screening, and pathology and specialist laboratory services [103]
Disparity and limited access to anesthetic care	High levels of morbidity and mortality are particularly prevalent in rural areas, which tend to be underequipped in both infrastructure and manpower. Difficulties of access to healthcare in fragile and marginalized areas are exacerbated by logistical, geographical, social, cultural, or financial barriers [103]. Many populations, especially in rural areas, do not have access to safe and reliable anesthesia services. Financial protection systems are often incomplete and inequitable [104]. The World Federation of Societies of Anesthetists reports that rural and remote areas with limited access to surgical treatment suffer mainly from a shortage of anesthesia professionals [95]
Lack of continuing education	Limited access to continuing education [95-97]. Variation in the quality of anesthetist training

These various challenges represent major obstacles that developing countries must overcome to improve anesthesia care for their populations.

Anesthesia services in low- and middle-income countries face major challenges. Lack of financial resources and adequate infrastructure leads to underinvestment due to limited healthcare budgets, while weak healthcare systems lead to inadequate care and poor management of anesthesia care, resulting in care gaps. However, in line with WHO’s policy guidance on strengthening health systems, a multidimensional approach is essential, and governments must increase funding for health systems by 12% of national budgets, as recommended by WHO, and 15%, as recommended by the Abuja-African Union statement [105]. This will enable investments in relevant medical infrastructure and equipment. However, the shortage of qualified anesthesiologists is also a concern, as most anesthesia procedures are performed by non-physicians. This situation is exacerbated by low pay and precarious working conditions, making it difficult to retain staff. Therefore, developing and strengthening human resources, improving the working environment, providing psychological support, and promoting further training are crucial. This approach is necessary to achieve WHO’s minimum threshold of 4.45 doctors and nurses per 1,000 inhabitants (44.5 per 10,000 people) by 2030. This target is consistent with achieving the health-related Sustainable Development Goals (SDGs), in particular, universal health coverage (UHC, SDG 3) and strengthening the health workforce (SDG 3c) [106]. In addition, initiatives such as the Lancet Commission have a target of 20 surgeons, anesthesiologists, and obstetricians per 100,000 people by 2030 [107]. To achieve this target, increasing funding for anesthesiologist training is essential. Inequalities in access to anesthesia care are particularly pronounced in rural areas, which are often poorly equipped and remote. Logistical, geographic, social, and cultural barriers limit access to vulnerable groups, resulting in many patients being unable to obtain safe anesthesia care. For achieving SDG target 3.8, it is crucial to ensure that all people benefit from universal health coverage, including protection from financial risk and access to quality essential health services, as well as safe, effective, quality, and affordable essential medicines and vaccines [108]. In conclusion, collaboration between governments, health organizations, and international actors is essential to addressing today’s challenges. It is crucial to develop sustainable solutions to improve the health and well-being of healthcare professionals and patients. However, a multifaceted approach combining efforts at local, national, and international levels will lead to more resilient healthcare systems and access to safe, quality anesthesia for all.

Future directions

The anesthesiology of the future will be marked by technological transformation, enabling personalized, optimized care. Indeed, AI, genomics, and digital simulation will offer a deeper understanding of each patient’s individual needs, leading to more precise and tailored interventions. However, the integration of these technologies raises significant challenges and will require considerable effort.

Simulation and Virtual Reality in Anesthesiology

Simulation is proving to be a valuable tool for developing research, enhancing professional performance, and improving patient safety. Moreover, anesthesiologists have been pioneers in the advancement of these technologies, developing interactive patient simulators, whether computer screens or sophisticated mannequins, or training programs for their use [109], enabling anesthesiologists to train in emergency management without endangering patients’ lives, and considerably improving their technical and non-technical skills, essential for effective and safe management [110]. Simulation also helps identify and resolve safety issues in anesthesia processes, by visualizing the consequences of decisions, and to better understand the causes. It also facilitates the exploration of interpersonal interactions in the clinical environment [111,112]. By reproducing realistic scenarios, virtual reality contributes to better preparation and training of trainees and offers them the opportunity to gain practical experience in a safe setting. Some studies have shown that integrating these technologies improves performance in real-life situations, reduces errors, and optimizes patient management [113].

Artificial Intelligence in Anesthesiology

AI plays an important and multifaceted role in improving anesthesia safety, as demonstrated by numerous recent studies [17]. Indeed, AI-based anesthesia decision support systems can analyze patient data, such as vital signs, physiological parameters, and drug information, thus optimizing the role of anesthesiologists by providing personalized recommendations and real-time alerts on critical changes for better patient management [17,114]. In addition, studies have shown that these systems reduce dosing errors and improve responsiveness to changes in the patient’s condition. AI-assisted feedback loops have been developed to optimize pharmacological and hemodynamic management during anesthesia [115]. Furthermore, AI is making significant progress in the field of anesthesiology. Indeed, ML techniques can analyze large amounts of data, identify trends, and predict outcomes, thus improving diagnosis, treatment, and interventions. Furthermore, several studies have shown that the AI methods tested generally outperform traditional methods in terms of performance. Table 2 presents AI systems that are promisingly integrated into clinical anesthesiology practice to improve anesthesia depth monitoring, anesthesia control, event and risk prediction, ultrasound guidance, pain management, and operating room organization [116,117].

**Table 2:** The use of artificial intelligence in anesthesia.

Features	Key benefits of artificial intelligence	Examples of effectiveness
Anesthesia depth monitoring	Better control of the depth of anesthesia. Meets the need for anesthesia depth monitoring in three distinct states. Induction of anesthesia. Maintenance of anesthesia. Recovery from anesthesia	Artificial intelligence-based anesthesia depth monitoring, achieving an accuracy of 94.1%, is better than all other comparative methods tested, while reducing costs [118]
Automated control system	More precise and stable administration of anesthetic drugs. Dose optimization. Reduced potential for human errors	Adequate anesthesia management for a significantly higher proportion of time (87.21%) than the traditional manual approach (65.19%). Adverse events were significantly less frequent in the group using the automated system (90.0% vs. 100.0%) [119]
Prediction of adverse events	Improved clinical decision-making and early identification of complications	Early prediction to prevent associated complications, a machine-learning algorithm can predict intraoperative hypotension 15 minutes before its onset, with a sensitivity of 88% and a specificity of 87% [120]
Ultrasound guidance	Improved accuracy, diagnostic efficiency, and practicality. Identification of anatomical structures. Automation of certain tasks	Successful identification of anatomical structures in 99.7% of cases and confirmation of the correct ultrasound view in 99.3% of analyses [121]
Pain management	Pain assessment. Pain prediction. Clinical decision-support. Pain self-management	Positive effect on pain recognition, prediction, and self-management [122]
Organization of the operating room	Better planning and allocation of resources. Optimization of critical care unit utilization	Significant impact on operating room management, improving procedure duration prediction, resource optimization, and cancellation detection [123]

However, despite these promising prospects, the adoption of AI in anesthesia is not yet widespread in practice. The use of these technologies raises ethical issues, particularly around the collection, validation, and transfer of patient data. As a result, clinicians need to understand the fundamentals of AI so that they can make the most of it to improve anesthesia care [124].

Genomics in Anesthesia

The integration of genomics in anesthesia offers many opportunities to improve patient care, ranging from risk prediction to treatment optimization. Indeed, genomics has a significant impact on anesthesiology by providing a better understanding of the genetic factors influencing patient response to anesthesia, the risk of perioperative complications, and clinical outcomes. In addition, genetic analysis provides a better understanding of individual variability in response to anesthetic agents and helps anticipate the risk of complications [125]. Pharmacogenomics also aims to understand how a patient’s genetic makeup influences drug action and side effects. Genetic factors can account for up to 20% of the total number of adverse reactions reported [126]. Pharmacogenetics also makes it possible to predict the efficacy and toxicity of anesthetic drugs based on the patient’s genetic profile, thereby improving the safety and efficacy of care [127]. Certain genetic variations may predispose patients to increased sensitivity to opioids, a higher risk of developing malignant hyperthermia in response to halogenated agents, or prolonged paralysis due to muscle relaxants; however, the identification of these genetic biomarkers makes it possible to tailor anesthetic management in a more personalized manner. In-depth knowledge of these genomic aspects enables anesthesia to be adapted in a more personalized way, with close monitoring and cautious use of certain drugs, to reduce risks in these fragile patients [128]. Indeed, thanks to genomics, anesthesiologists can envisage a more personalized approach by adapting anesthetic strategies according to the patient’s genetic profile. This can lead to better pain management, fewer side effects from anesthetic drugs, and optimized surgical outcomes. In addition, genomics opens the way to advances in predicting anesthesia-related risks, preventing complications, and improving overall perioperative care [125]. On the other hand, several studies have shown that genetic variations influence the metabolization of anesthetics, which can lead to varied responses, ranging from optimal efficacy to severe adverse effects [129]. Table 3 highlights the applications of pharmacogenetics, pharmacogenomics, and disease genomics in anesthesia, detailing the functionalities of these disciplines and highlighting their advantages and effectiveness with examples.

**Table 3:** The contribution of genomics to anesthesia practice.

Features	Benefits	Examples of efficacy
Pharmacogenetics and pharmacogenomics	Identification of the impact of genetic variants on drug dosage, response, metabolism, and safety. Dose personalization. Optimization of anesthesia administration and patient outcomes. Reduction of the risks of anesthesia	Pharmacogenomic testing before anesthesia can suggest dose adjustments, detect responder and non-responder patients, and predict the risk of certain adverse effects [129]. Pharmacogenetics optimizes diazepam dosage and minimizes the risk of toxicity. However, slow metabolizers of CYP2C19 and CYP2B6 require dose reductions ranging from 25% to 70% to avoid excessive drug exposure [130,131]
Disease genomics	Identification of genetic risk factors. Adaptation of anesthetic protocols to genetic diseases	Patients with myasthenia gravis are more sensitive to sedatives and anesthetics, e.g., high-dose benzodiazepines and opioids may cause respiratory depression [132]

Robotics in Anesthesia

Robotic anesthetists represent a significant advancement in the medical field by being able to perform a variety of tasks autonomously or semi-autonomously, such as monitoring patients’ vital signs, controlled administration of anesthetic drugs, and airway management [133,134].

Indeed, the integration of robotics in anesthesia represents a promising advancement in the medical field, offering the possibility of automating certain repetitive and risky tasks while providing clinical decision support, as well as allowing robotics to combine different functions to create autonomous systems that can be divided into three categories. These include pharmacological to calculate and administer drug doses, and mechanical to imitate manual and cognitive gestures to aid clinical decision-making. This considerably reduces the risk of human error and ensures greater consistency in anesthetic care. In addition, the use of robots enables anesthetists to concentrate on more complex tasks requiring greater clinical judgment, improving the quality of care.

On the other hand, tele-anesthesia offers the possibility of providing safe anesthetic care when qualified personnel are unavailable or require assistance. Mechanical robots are being developed [133] and significant progress has been made in the field of anesthetic robotics, such as in drug delivery and management of patient physiological parameters. For instance, pharmacological robots have been successfully used to control conscious sedation, manage hemodynamic status, and administer anesthesia while maintaining specific parameters in target situations. Innovation in the field of anesthetic robotics seems necessary to protect medical staff from infection, particularly by avoiding proximity to patient airways [135]. Table 4 presents a classification of robotic systems used in anesthesia. It describes the functionalities of each category, highlighting their advantages supported by studies and evidence of effectiveness.

**Table 4:** The role of robotics in anesthesia.

Robot categories	Functionality	Benefits	Studies and examples	Evidence of efficacy
Pharmacological robots	Automated anesthetic drug titration (closed loop)	Greater precision in drug administration. Reduction in interindividual variations. Optimization of the depth of anesthesia	BIS (Absalom et al., 2003 [136]). CLADS, McSleepy (Hemmerling and Terrasini, 2012 [133])	Reduction in adverse events, improved postoperative recovery, and assistance in administering medication
Mechanical robots	Assistance with manual procedures (intubation, nerve blocks)	The Kepler intubation system in 2012 (91% success rate achieved in 93 seconds). The automated intubation robotic system (AIRS) was recently implemented during the coronavirus pandemic to safely intubate patients while shielding the medical personnel operating from a distance and preventing contamination [137]. Greater precision, reduced risk of complications, and improved visualization	Kepler intubation system (Hemmerling et al., 2012 [138]). Magellan system will enable faster acquisition of nerve block needle guidance skills than the traditional manual method (Morse et al., 2013 [139])	Allows the video laryngoscope to be positioned precisely to facilitate intubation. Remote operation, stabilization of hand movements, the possibility of freeing the user’s hands, and performance of anesthesiologists’ tasks
Decision-support systems and teleoperation	Provision of real-time clinical information. Detection of adverse events and teleoperation	Improved clinical decision-making. Reduced medical errors and remote access to expertise	Automated intubation robotic system (AIRS) (Gupta et al., 2023 [140]). Teleoperation in anesthesia [137]	Reducing undesirable events (improving the quality of care). Telemedicine and teleoperation have the potential to facilitate access to specialist care for underserved rural and urban populations

However, despite these advances, the widespread integration of anesthesia robots into clinical practice remains an elusive goal, requiring further research, testing, and improvements to ensure their functionality, reliability, and safety.

Benefits and limitations of innovations in anesthesiology

Emerging Technological Opportunities

New technologies are profoundly transforming the field of anesthesia, providing innovative solutions to the persistent challenges facing the practice itself, patients, practitioners, and low- and middle-income countries. Creating realistic virtual environments enables professionals to train repeatedly, acquire technical skills, and develop their ability to manage complex situations with complete safety. It also promotes collaborative learning and improved communication skills. AI is revolutionizing decision-making in anesthesia. By analyzing large quantities of data, AI can help predict risks, optimize drug dosages, and personalized care. It can also detect complications more quickly and improve patient monitoring, helping to avoid errors and mistakes. Limited human performance, pain management, and the organization of the operating room checklist ensure that the anesthetic procedure runs smoothly. Furthermore, genomics opens the way to precision medicine in anesthesia by identifying individual genetic variations and pinpointing patients likely to develop complications. Thanks to pharmacogenetics, pharmacogenomics, and the patient’s genome, it is possible to personalize anesthetic care, adapt anesthetic protocols, and reduce the risk of complications. This personalized approach improves treatment efficiency and optimizes patient safety. Finally, robotics offers new perspectives in terms of surgical assistance and patient monitoring. Pharmacological robots help with drug titration and optimization, while mechanical robot systems can perform repetitive tasks with great precision, reducing medical staff fatigue, freeing up anesthetists to perform and concentrate on other tasks, and improving the quality of care. Telemedicine also facilitates access to care in rural and underserved areas.

Table 5 highlights the direct impact of new technologies on the current challenges in anesthesiology. It highlights how these innovations are helping overcome the obstacles encountered in this field, optimizing patient safety, the efficiency of procedures, and the training of professionals.

**Table 5:** Direct impact of emerging technologies on current challenges in anesthesiology.

Issues	Current situation	Opportunities offered by new technologies
Risks associated with anesthetic procedures	Risks associated with anesthetic agents (side-effects, serious complications).	Artificial intelligence (AI) has been developed to analyze large quantities of data, improve clinical decision-making, optimize dosing through an automated control system for more precise and stable administration of anesthetic drugs, predict adverse events, improve clinical decision-making, early identification of complications, optimizing pharmacological and hemodynamic management during anesthesia to reduce dosage errors and anticipating the adverse effects of anesthetic agents. Genomics makes it possible to predict risks and optimize treatments by analyzing the genetic factors that influence patient response, the risk of perioperative complications, and clinical outcomes. Pharmacogenetics assesses the efficacy and toxicity of anesthetic drugs according to the patient’s genetic profile, thereby contributing to personalized care. Robotics enables the controlled administration of anesthetic drugs via pharmacological robots, which have been successfully used to manage conscious sedation and hemodynamic status. It also facilitates automated closed-loop titration of anesthetics, maintaining specific parameters in target situations
	Technical problems with anesthetic procedures and practice (failed intubation, etc.)	Mechanical robots assist in manual procedures such as intubation and nerve blocks, performing repetitive tasks with great precision
	Complications of locoregional anesthesia (nerve damage, toxicity, etc.)	AI facilitates ultrasound guidance, improving diagnostic accuracy and efficiency while identifying anatomical structures and automating certain tasks
Patient-related risks	Factors and medical history of patients that increase the risk. Interactions of medications taken by patients with the anesthetic agents administered	AI can predict adverse events, improve clinical decision-making, and identify complications at an early stage. By analyzing large quantities of data, AI improves diagnosis, treatment, and interventions. Genomics enables a personalized approach to anesthesia by adapting treatments to genetic factors while monitoring the use of drugs to minimize risks. It also facilitates the identification of genetic risk factors and the adjustment of anesthetic protocols according to genetic diseases
Risks associated with healthcare professionals	Anxiety, exhaustion, workload, and fatigue	Robotics, by integrating pharmacological solutions for calculating and administering drug doses, as well as mechanical systems that mimic manual and cognitive gestures, helps clinical decision-making. This reduces fatigue among medical staff, frees up anesthesiologists for other tasks, and improves the quality of care
	Reduced performance can lead to errors caused by incompetence, inappropriate decisions, or poorly executed actions. Failure to follow good practice can also lead to adverse events. Fear of legal action and the adoption of defensive medical practices can impact anesthetic practice and have repercussions for patients	Simulation enhances professional performance by training in patient-safe emergency management, identifying safety issues in anesthesia, and visualizing the consequences of decisions, thereby reducing errors and optimizing patient management. In addition, virtual reality offers trainees a safe hands-on experience, helping reduce errors and improve patient management. AI, through anesthesia decision-support systems, analyzes patient data, including vital signs and medication information, to provide personalized recommendations. It also optimizes resource planning, improves diagnosis by identifying trends, and ensures better control of the depth of anesthesia at all stages. Robotics: Decision-support systems provide real-time clinical information and detect adverse events
	Interpersonal communication problems within groups	Simulation facilitates the exploration of interpersonal interactions in a clinical setting and encourages collaborative learning, thereby improving communication skills
Challenges specific to low- and middle-income countries	Lack of infrastructure and limited resources, as well as shortage of care staff and workload. Fragile systems and inadequate provision. Disparity and limited access to anesthetic care. Insufficient continuing training	AI enables efficient organization of the operating theater, improves resource planning and allocation, and optimizes the use of care units. Telemedicine extends the coverage of care and facilitates access in rural and underserved areas. Robots allow anesthesiologists to concentrate on more complex tasks by providing assistance that reduces workload and ensures continuous monitoring of vital signs for the early detection of abnormalities. Remote anesthesia enables safe anesthetic care to be delivered where there are no qualified staff or where assistance is required, particularly in underserved areas. Simulation is the use of interactive patient simulators, whether in the form of computer screens or sophisticated mannequins, as well as initial and continuing training programs, which are valuable tools for developing research, improving professional performance, and enhancing patient safety

These technologies open up exciting new prospects for anesthesia. By refining diagnoses, personalizing treatments, optimizing processes, improving training, and enhancing patient safety, they are helping to shape safer, more efficient anesthetic practices. Continued research and development in this field are essential to further advance anesthesia care.

Ethical Limitations and Considerations

New technologies offer many opportunities to improve the safety, efficiency, and personalization of anesthesia care. However, their deployment raises significant challenges, such as the lack of quality data, algorithmic biases that could mislead diagnosis and results, ethical issues concerning confidentiality, liability in the event of medical error, inequality of access due to high cost, and the absence of a legal framework to frame their use. To overcome these challenges, it is essential to develop more transparent algorithms, clean up data, closely involve clinicians, and establish clear regulatory frameworks. In this way, the successful integration of these technologies at the service of healthcare can profoundly transform the practice of anesthesia [14,117,128,141-143]. Table 6 highlights the different factors to be taken into account when dealing with innovations in anesthesiology; identifying issues relating to data, techniques, ethics, socioeconomic aspects, regulations, and human resources; identifying the main challenges to be met; and providing concrete examples, while proposing recommendations. This multidimensional approach underlines the need for a global and in-depth reflection on the potential impacts of these innovations, beyond mere technical benefits, to anticipate and responsibly manage the limits and ethical considerations associated with the deployment of these new solutions in anesthesiology.

**Table 6:** Challenges and prospects for emerging technologies.

Dimension	Key challenges and limits	Consequences	Recommendations
Data	Quality, quantity, and bias: The data used to train artificial intelligence (AI) models must be of high quality, representative of the population, and free from bias. Medical data is often heterogeneous, incomplete, and may contain biases related to patient selection or measurement instruments. Confidentiality: The protection of patients’ data is essential to respect privacy and avoid misuse	Misdiagnosis, inadequate treatment, and discrimination	Standardized data formats, rigorous cleansing and validation, anonymization, and enhanced protection through security measures (encryption, restricted access)
Techniques	Explicability: AI models and intense neural networks are often regarded as “black boxes,” making it difficult to interpret their decisions. Algorithmic biases: Biases present in training data can be amplified in models, leading to discrimination. Generalization: AI models may need help to generalize findings to new situations or patients	Lack of trust in healthcare professionals, erroneous medical decisions, and loss of patient autonomy	Development of explicability techniques (LIME, SHAP), cross-validation, testing on a variety of data, and close collaboration between computer scientists and clinicians
Ethics	Responsibility: In the event of a medical error involving AI, it is difficult to determine who is responsible (doctor, IT specialist, manufacturer). Justice: The use of AI in healthcare can accentuate inequalities in access to care if it is not developed and deployed equitably. Autonomy: AI can challenge patient autonomy in decision-making	Discrimination, infringement of fundamental rights	Drawing up codes of conduct, clear regulations, ethics committees, and raising awareness among professionals
Socioeconomic	Costs: AI systems can be costly to develop, implement, and maintain, limiting access to healthcare facilities with fewer resources. Access: Access to AI can be uneven, creating disparities in the quality of care. Employment: The automation of certain tasks by AI may lead to job losses in the healthcare sector	The digital divide, widening social inequalities, technological unemployment	Development of affordable solutions, public support policies, and ongoing training for professionals
Regulations	Uncertain legal framework: The legal framework governing the use of AI in healthcare is still under construction, creating uncertainties about liability in the event of an incident	Legal uncertainties, brakes on innovation	Clarification of the legal framework and specific insurance for healthcare facilities using the new technology
Human resources	Resistance to change, lack of training, fear of job loss	Loss of skills, demotivation, recruitment difficulties, user errors	Ongoing training, support for change, development of user-friendly interfaces, promotion of healthcare professions

## Conclusions

Anesthesiology has made remarkable progress in recent years, owing to major advances in practice, technology, and patient safety. However, many challenges remain, and to overcome them, several measures and recommendations are essential, including the integration of new technological innovations. However, further empirical studies and rigorous experiments are needed to address the associated technical, ethical, and regulatory issues. By investing in research, encouraging collaboration between the various players in the field, and adopting a culture of continuous improvement, anesthesia can continue to progress and offer increasingly safe and equitable care to the global population.
